# Immune-mediated inflammatory diseases and leukocyte telomere length: A Mendelian randomization study

**DOI:** 10.3389/fgene.2023.1129247

**Published:** 2023-04-17

**Authors:** Meiling Liu, Ping Luo, Lihua Liu, Xianping Wei, Xue Bai, Jicui Li, Linlin Wu, Manyu Luo

**Affiliations:** Department of Nephrology and Rheumatology, The Second Hospital of Jilin University, Changchun, China

**Keywords:** Mendelian randomization, telomere length, immune-mediated inflammatory diseases, genetic variants, causal effect

## Abstract

**Objective:** To elucidate the potential causality of leukocyte telomere length (LTL) with immune-mediated inflammatory diseases (IMIDs), we conducted a Mendelian randomization (MR) study.

**Methods:** The genetically predicted causation between LTL and IMIDs was evaluated using a two-sample MR method. We analyzed 16 major IMIDs, which included systemic lupus erythematosus (SLE), inflammatory bowel disease (IBD), ulcerative colitis (UC), Crohn’s disease (CD), ankylosing spondylitis (AS), sicca syndrome (SS), rheumatoid arthritis (RA), type 1 diabetes (T1D), primary sclerosing cholangitis (PSC), idiopathic pulmonary fibrosis (IPF), atopic dermatitis (AD), sarcoidosis, hypothyroidism, hyperthyroidism, psoriasis, and childhood asthma. The random-effects inverse-variance weighted (IVW) method was performed as the main analytical approach in MR. Various sensitivity analyses, including MR-Egger, MR robust adjusted profile score (MR-RAPS), weighted median, MR pleiotropy residual sum and outlier (MR-PRESSO) methods, weighted mode, radial plot, and radial regression, were used to guarantee the robustness of the results and detect horizontal pleiotropy. Cochran’s Q value was calculated to check for heterogeneity, and the MR Steiger approach was used to test the causal direction.

**Results:** The MR results indicated significant inverse associations of LTL with risks of psoriasis (OR: 0.77, 95% CI: 0.66–0.89, and *p* = 3.66 × 10^−4^), SS (OR: 0.75, CI: 0.58–0.98, and *p* = 0.03), RA (OR: 0.77, 95% CI: 0.68–0.88, and *p* = 9.85 × 10^−5^), hypothyroidism (OR: 0.84, 95% CI: 0.78–0.91, and *p* = 7,08 × 10^−6^), hyperthyroidism (OR: 0.60, 95% CI: 0.44–0.83, and *p* = 1.90 × 10^−3^), sarcoidosis (OR: 0.67, 95% CI: 0.54–0.83, and *p* = 2.60 × 10^−4^), and IPF (OR: 0.41, 95% CI: 0.29–0.58, and *p* = 4.11 × 10^−7^) in the FinnGen study. We observed that longer LTL was associated with an increased risk of AS susceptibility (OR: 1.51, 95% CI: 1.18–1.94, and *p* = 9.66 × 10^−4^). The results of the IVW method showed no causal relationship between TL and SLE (OR: 0.92, 95% CI: 0.62–1.38, and *p* = 0.69) in the FinnGen study; however, a significantly positive correlation was shown between LTL and SLE in another larger GWAS (OR: 1.87, 95% CI: 1.37–2.54, and *p* = 8.01 × 10^−5^).

**Conclusion:** Our findings reveal that abnormal LTL has the potential to increase the risk of IMIDs. Therefore, it could be treated as a predictor and may provide new potential treatment targets for IMIDs. However, the change of LTL may not be the direct cause of IMIDs. Further studies should aim at the pathogenic mechanism or potential protective effects of LTL in IMIDs.

## Introduction

IMIDs are a clinically heterogeneous group that is characterized by disorders of the immune system that trigger chronic inflammation in any organ system (Forbes et al., 2018). IMIDs include SLE, SS, RA, IBD, AS, sarcoidosis, cutaneous inflammatory conditions such as psoriasis and atopic dermatitis, asthma, and autoimmune endocrine diseases, such as hyperthyroidism and hypothyroidism. Different IMIDs show unrelated clinical manifestations. Although they share common inflammatory pathways (Forbes et al., 2018; Fitzgerald et al., 2021), the etiology of these conditions remains poorly understood. In addition, the prevalence of IMIDs in western populations is approximately 7% (Bayry and Radstake, 2013). It leads to considerable socioeconomic burden and impairs the quality of patients’ lives. Thus, it is urgent to identify the causative factors to elucidate biological mechanisms that could be conducive in finding potential therapeutic targets.

Telomeres are special protein–DNA structures at the ends of eukaryotic linear chromosomes that are composed of tandem short fragment repeats (TTAGGG) and some telomere-binding proteins (Blackburn et al., 2015). The functioning of TL plays a significant role in maintaining chromosome integrity and stability during cellular divisions (Blackburn et al., 2015; Turner et al., 2019). In most somatic tissues, telomere repeat sequences are shortened once after each cell division. When a critically short TL is reached, it will ultimately trigger cellular senescence, cell cycle arrest, and apoptosis (Heba et al., 2021). Therefore, the length of the telomere is viewed as a “molecular clock” for biological aging. During recent decades, a close association between LTL and IMIDs has been reported (Steer et al., 2007; Tamayo et al., 2010; Coussens et al., 2016; Fessler et al., 2016; Newton et al., 2019), indicating the underlying involvement of TL in pathogenesis of IMIDs. A noticeable difference in the alteration of TL in IMID patients when compared to controls has been suggested by recent studies, which have shown mixed results. A study observed longer TL in patients with AS than in the control (Tamayo et al., 2010), while another study revealed the reverse results (Tamayo et al., 2014; Fessler et al., 2016). Furthermore, an observational study found AD patients have shorter TL (Wu et al., 2000), but another finding showed the shortening of LTL did not promote AD development until 1 year of age (Suh et al., 2019b).

However, the statistical power of these studies may not be strong because of the vulnerability of reverse causation and residual confoundings. Owing to the limitations of a previous study, a novel and powerful analytical technique—the two-sample MR (Davies et al., 2018)—was implemented to investigate the causal association of exposure (LTL) on outcomes (IMIDs). The distribution of alleles is random at conception following Mendel’s laws of inheritance and generally not likely to be influenced by reverse causation or confounding bias. Therefore, under specific assumptions, MR is credible for elucidating the causality between LTL and IMIDs.

## Method and materials

### Study design

**FIGURE 1:**
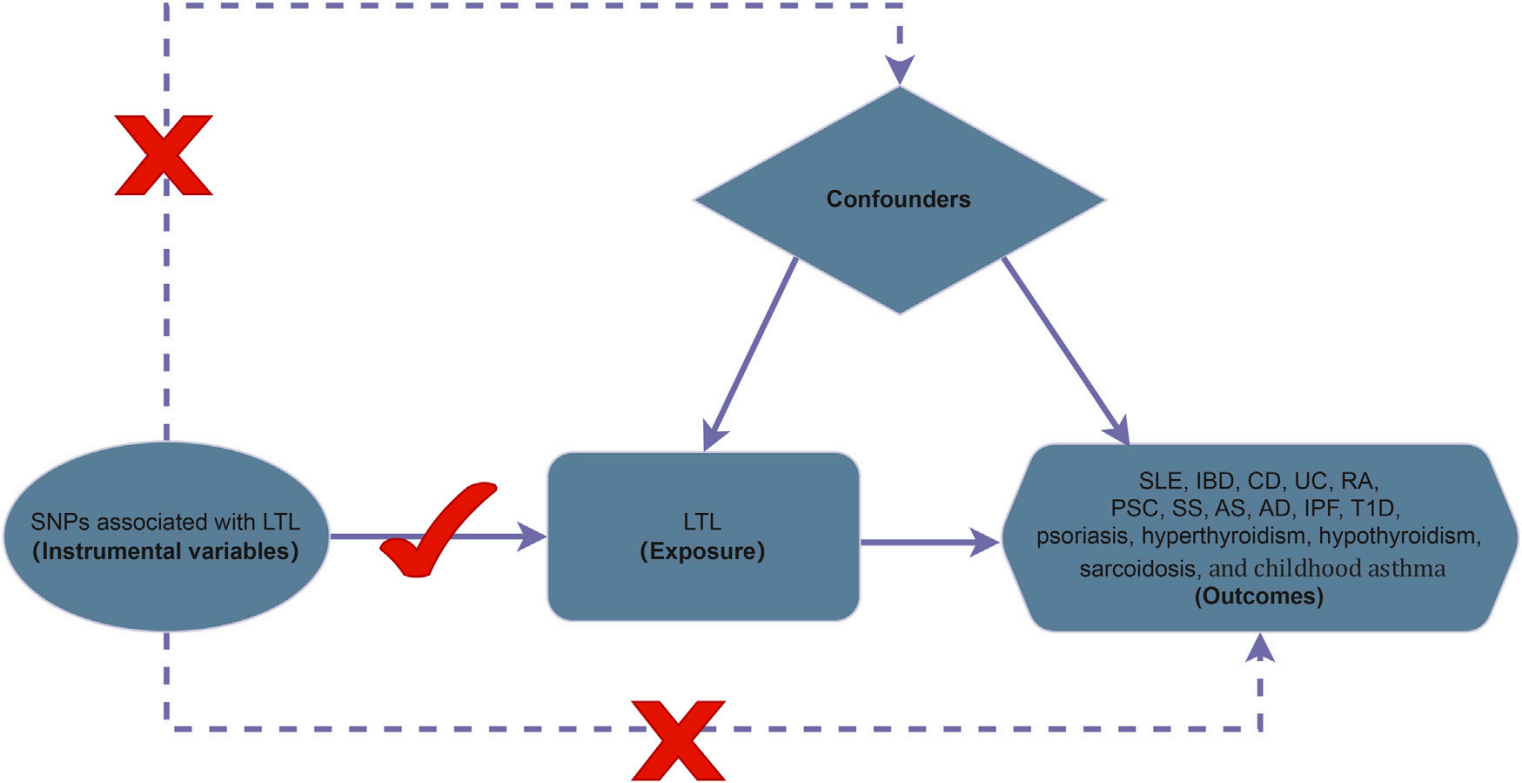
The framework of two sample Mendelian randomization analyses. Mendelian randomization model of LTL and risk of IMIDs. MR analysis can provide valid causality when three core assumptions are satisfied as follows: 1) the selected instruments of genetic variations are robustly associated with the exposure (LTL). 2) The selected instruments of SNPs should not be linked to any potential confounders of the relationship between exposure and outcomes. 3) The selected instruments of genetic variants should have no direct correlation with the outcome by any other causal pathway except through the way of exposure. SNP, single nucleotide polymorphism; LTL, leukocyte telomere length; MR, Mendelian randomization; SLE, systemic lupus erythematosus; IBD, inflammatory bowel disease; UC, ulcerative colitis; CD, Crohn’s disease; AS, ankylosing spondylitis; SS, sicca syndrome; RA, rheumatoid arthritis; T1D, type 1 diabetes; PSC, primary sclerosing cholangitis; IPF, idiopathic pulmonary fibrosis; AD, atopic dermatitis.

For the study design, we performed a two-sample MR to investigate the association of LTL with IMIDs, which included SLE, IBD, CD, UC, RA, PSC, SS, AS, AD, IPF, T1D, psoriasis, hyperthyroidism, hypothyroidism, sarcoidosis, and childhood asthma. We utilized most of the summary data of IMIDs, which do not overlap with those of the GWAS of LTL. Then, we conducted a two-sample MR and several sensitivity analyses to identify the causal effect (Figure 1).

### GWAS data of leukocyte telomere length

Genetic association data for the LTL were retrieved from a GWAS of 472,174 European participants (Codd et al., 2021). All participants in the UK Biobank were aged 40–69 years, with a similar proportion of males (45.8%) and females (54.2%). The measurements of LTL were obtained by using an established quantitative PCR assay and were subjected to several quality checks to control and adjust for ethnicity, gender, age, and technical factors, as described in a previous study.

### GWAS data of multiple IMIDs

We used publicly available GWAS summary statistics for different IMIDs acquired from the FinnGen Consortium. The release of R7 GWAS of the FinnGen consortium data was used (https://r7.finngen.fi/). SNPs were analyzed by performing a mixed-model logistic regression model, adjusting for sex, age, 10 principal components (PCs), genetic relatedness, and genotyping batch. Detailed information on it can be found on the official website (https://www.finngen.fi/en). The number of cases and controls is presented in Tables 1, 2. Another SLE summary statistic was acquired from the recent meta-analysis of 5,201 patients and 9,066 controls of European descent in this study (Bentham et al., 2015).

**TABLE 1:** Two-sample Mendelian randomization estimations showing no causal effect of leukocyte telomere length on immune-mediated inflammatory diseases.

Methods	Outcome	Cases/controls	OR	95% CI (low)	95% CI (up)	*p*-value	Ph	Q-statistics
Inverse-variance weighted	SLE (Finn)	835/300,162	0.92	0.62	1.38	0.69	1.00	77.13
MR-Egger			1.10	0.55	2.22	0.78	0.99	
Robust adjusted profile score (RAPS)			0.93	0.62	1.42	0.75		
Weighted median			1.25	0.67	2.32	0.48		
Weighted mode			1.44	0.65	3.18	0.37		
MR-PRESSO			NA	NA	NA	NA		
MR-Egger intercept			−0.006			0.53		
Inverse-variance weighted	Ulcerative colitis (all Crohn cases excluded)	3,407/303,191	1.13	0.92	1.40	0.25	0.9	90.75
MR-Egger			1.09	0.74	1.59	0.67	0.89	
Robust adjusted profile score (RAPS)			1.13	0.91	1.40	0.29		
Weighted median			1.03	0.73	1.45	0.88		
Weighted mode			0.98	0.67	1.44	0.93		
MR-PRESSO			NA	NA	NA	NA		
MR-Egger intercept			0.001			0.81		
Inverse-variance weighted	Crohn’s disease (all ulcerative colitis excluded)	1,021/301,234	0.93	0.65	1.35	0.71	0.88	91.31
MR-Egger			0.87	0.46	1.64	0.66	0.86	
Robust adjusted profile score (RAPS)			0.94	0.64	1.37	0.74		
Weighted median			0.80	0.45	1.43	0.45		
Weighted mode			0.87	0.43	1.75	0.7		
MR-PRESSO			NA	NA	NA	NA		
MR-Egger intercept			0.003			0.78		
Inverse-variance weighted	Crohn’s disease (small intestine)	1,501/296,008	1.24	0.91	1.68	0.17	0.97	81.94
MR-Egger			1.02	0.61	1.73	0.93	0.96	
Robust adjusted profile score (RAPS)			1.22	0.89	1.67	0.22		
Weighted median			1.11	0.69	1.77	0.67		
Weighted mode			1.07	0.63	1.83	0.80		
MR-PRESSO			NA	NA	NA	NA		
MR-Egger intercept			0.007			0.39		
Inverse-variance weighted	Crohn’s disease (large intestine)	1,220/296,008	0.79	0.56	1.11	0.17	0.95	85.31
MR-Egger			0.88	0.49	1.56	0.66	0.95	
Robust adjusted profile score (RAPS)			0.79	0.56	1.12	0.19		
Weighted median			1.01	0.61	1.67	0.98		
Weighted mode			0.99	0.57	1.70	0.97		
MR-PRESSO			NA	NA	NA	NA		
MR-Egger intercept			−0.004			0.67		
Inverse-variance weighted	Type 1 diabetes	8,671/255,466	1.01	0.88	1.15	0.91	0.87	89.75
MR-Egger			1.04	0.82	1.30	0.76	0.86	
Robust adjusted profile score (RAPS)			1.03	0.89	1.18	0.71		
Weighted median			1.01	0.83	1.24	0.91		
Weighted mode			1.03	0.82	1.30	0.80		
MR-PRESSO			NA	NA	NA	NA		
MR-Egger intercept			−0.001			0.77		
Inverse-variance weighted	Primary sclerosing cholangitis	1,231/273,442	1.08	0.77	1.52	0.65	1.00	69.08
MR-Egger			1.00	0.56	1.80	0.99	1.00	
Robust adjusted profile score (RAPS)			1.08	0.76	1.53	0.66		
Weighted median			0.96	0.58	1.59	0.87		
Weighted mode			0.95	0.50	1.82	0.88		
MR-PRESSO			NA	NA	NA	NA		
MR-Egger intercept			0.003			0.75		
Inverse-variance weighted	Inflammatory bowel disease	8,704/300,450	1.13	0.99	1.29	0.07	0.84	86.74
MR-Egger			1.16	0.92	1.46	0.22	0.83	
Robust adjusted profile score (RAPS)			1.13	0.98	1.30	0.08		
Weighted median			1.07	0.88	1.30	0.48		
Weighted mode			1.10	0.89	1.37	0.37		
MR-PRESSO			NA	NA	NA	NA		
MR-Egger intercept			−0.001			0.80		
Inverse-variance weighted	Atopic dermatitis (strict definition)	7,895/300,873	0.95	0.82	1.09	0.45	0.94	83.98
MR-Egger			1.04	0.80	1.34	0.79	0.94	
Robust adjusted profile score (RAPS)			0.94	0.81	1.09	0.39		
Weighted median			0.96	0.77	1.20	0.74		
Weighted mode			0.94	0.72	1.21	0.63		
MR-PRESSO			NA	NA	NA	NA		
MR-Egger intercept			−0.003			0.42		
Inverse-variance weighted	Childhood asthma (age <16 years)	4,549/175,182	0.94	0.78	1.14	0.53	0.94	87.39
MR-Egger			0.98	0.70	1.36	0.90	0.93	
Robust adjusted profile score (RAPS)			0.94	0.77	1.14	0.52		
Weighted median			1.01	0.75	1.35	0.96		
Weighted mode			1.04	0.72	1.49	0.85		
MR-PRESSO			NA	NA	NA	NA		
MR-Egger intercept			−0.001			0.78		

**TABLE 2:** Two-sample Mendelian randomization estimations showing the potential causality of leukocyte telomere length on immune-mediated inflammatory diseases.

Methods	Outcome	Cases/controls	OR	95% CI (low)	95% CI (up)	*p*-value	Ph	Q-statistics
Inverse-variance weighted	SLE (James Bentham)	5,201/9,066	1.87	1.37	2.54	8.01 × 10^−5^	0.76	69.02
MR-Egger			1.61	0.89	2.89	0.12	0.74	
Robust adjusted profile score (RAPS)			1.93	1.38	2.71	1.27 × 10^−4^		
Weighted median			1.86	1.14	3.03	0.01		
Weighted mode			2.10	1.05	4.23	0.04		
MR-PRESSO			NA	NA	NA	NA		
MR-Egger intercept			0.005			0.56		
Inverse-variance weighted	Ankylosing spondylitis	2,252/227,388	1.51	1.18	1.94	9.66 × 10^−4^	0.84	98.29
MR-Egger			1.58	1.03	2.43	0.04	0.82	
Robust adjusted profile score (RAPS)			1.52	1.17	1.97	1.53 × 10^−3^		
Weighted median			1.71	1.16	2.50	6.15 × 10^−3^		
Weighted mode			1.63	1.05	2.53	0.03		
MR-PRESSO			NA	NA	NA	NA		
MR-Egger intercept			−0.002			0.81		
Inverse-variance weighted	Psoriasis	6,995/299,128	0.77	0.66	0.89	3.66 × 10^−4^	0.44	104.44
MR-Egger			0.86	0.67	1.11	0.26	0.45	
Robust adjusted profile score (RAPS)			0.77	0.66	0.91	1.52 × 10^−3^		
Weighted median			0.76	0.61	0.96	0.02		
Weighted mode			0.79	0.61	1.04	0.10		
MR-PRESSO			NA	NA	NA	NA		
MR-Egger intercept			−0.004			0.25		
Inverse-variance weighted	Sicca syndrome	1981/300,162	0.75	0.58	0.98	0.03	0.99	77.55
MR-Egger			0.67	0.43	1.06	0.09	0.99	
Robust adjusted profile score (RAPS)			0.75	0.57	0.98	0.03		
Weighted median			0.75	0.50	1.12	0.16		
Weighted mode			0.72	0.42	1.22	0.23		
MR-PRESSO			NA	NA	NA	NA		
MR-Egger intercept			0.004			0.55		
Inverse-variance weighted	Rheumatoid arthritis	9,855/202,617	0.77	0.68	0.88	9.85 × 10^−5^	0.74	94.19
MR-Egger			0.74	0.59	0.93	0.01	0.73	
Robust adjusted profile score (RAPS)			0.76	0.66	0.87	6.23 × 10^−5^		
Weighted median			0.71	0.58	0.86	5.79 × 10^−4^		
Weighted mode			0.70	0.52	0.93	0.02		
MR-PRESSO			NA	NA	NA	NA		
MR-Egger intercept			0.002			0.62		
Inverse-variance weighted	Hypothyroidism	33,422/227,415	0.84	0.78	0.91	7,08 × 10^−6^	0.78	83.01
MR-Egger			0.88	0.77	1.00	4.77 × 10^−2^	0.78	
Robust adjusted profile score (RAPS)			0.84	0.78	0.91	7.94 × 10^−6^		
Weighted median			0.88	0.78	0.99	0.03		
Weighted mode			0.86	0.75	0.99	0.03		
MR-PRESSO			NA	NA	NA	NA		
MR-Egger intercept			−0.001			0.44		
Inverse-variance weighted	Hyperthyroidism	1,421/231,654	0.60	0.44	0.83	1.90 × 10^−3^	0.99	75.83
MR-Egger			0.55	0.32	0.96	0.04	0.99	
Robust adjusted profile score (RAPS)			0.60	0.43	0.83	2.23 × 10^−3^		
Weighted median			0.60	0.37	0.98	0.04		
Weighted mode			0.52	0.30	0.91	0.02		
MR-PRESSO			NA	NA	NA	NA		
MR-Egger intercept			0.003			0,71		
Inverse-variance weighted	Sarcoidosis	3,103/304,494	0.67	0.54	0.83	2.60 × 10^−4^	0.99	74.85
MR-Egger			0.69	0.48	1.00	0.05	0.99	
Robust adjusted profile score (RAPS)			0.66	0.53	0.82	2.47 × 10^−4^		
Weighted median			0.62	0.44	0.87	6.39 × 10^−3^		
Weighted mode			0.68	0.46	0.99	4.71 × 10^−2^		
MR-PRESSO			NA	NA	NA	NA		
MR-Egger intercept			−0.001			0.83		
Inverse-variance weighted	Idiopathic pulmonary fibrosis	1,514/306,063	0.41	0.29	0.58	4.11 × 10^−7^	1.00	66.11
MR-Egger			0.26	0.13	0.50	1.23 × 10^−4^	1.00	
Robust adjusted profile score (RAPS)			0.41	0.28	0.58	1.03 × 10^−6^		
Weighted median			0.32	0.19	0.54	2.45 × 10^−5^		
Weighted mode			0.28	0.13	0.60	1.43 × 10^−3^		
MR-PRESSO			NA	NA	NA	NA		
MR-Egger intercept			0.014			0.12		

### SNP selection

We retrieved SNPs of LTL as instruments, which are associated with LTL at a genome-wide significance threshold of *p* < 5 × 10^−8^. Then, to avoid colinearity between SNPs, we employed the remaining SNPs at a threshold of linkage disequilibrium clumping r^**2**^ < 0.001 exceeding a 10 Mb window using the European sample of 1,000 genome data as a reference panel. For the sake of accuracy and consistency in SNPs selected as IVs across different analyses, the SNPs were not replaced with proxy SNPs if they were missing variants for the outcome in the GWAS. In addition, palindromic SNPs with intermediate allele frequencies were excluded when we conducted a harmonization process between effect alleles for SNP exposure and variant alleles for effect estimates from the different data sets of outcome according to the same effect alleles. A total of 153 SNPs were obtained for leukocyte telomere length. We then calculated the F-statistic value of each SNP to evaluate its strength. SNPs with F values greater than 10 indicated strong instruments that could avoid weak instrument bias (Burgess et al., 2016). The F values of 153 SNPs in this study were above 10.

### Statistical analysis

For MR estimates, the IVW method was conducted as the main statistical analysis method. The Wald ratio estimates for each SNP were meta-analyzed under a random-effects model IVW. Given that this method can return unbiased estimates of a causal effect only if all SNPs have no invalid IV and unbalanced horizontal pleiotropy (Hartwig et al., 2017), we performed complementary sensitivity analyses, which included the MR-Egger, MR-RAPS, weighted median, weighted mode, and MR-PRESSO methods, to investigate the sensitivity and examine the robustness of the causal effects of LTL on the risk of each of the IMIDs. If the instrument strength independent of direct effect (INSIDE) assumption is satisfied, the MR-Egger method can examine whether the horizontal pleiotropic effects were presented in the data among all SNPs *via* the intercept and can also provide a reliable and unbiased assessment of causation (Bowden et al., 2015). An intercept value of the regression line that deviates remarkably from zero (*p*-value < 0.05) identifies horizontal pleiotropy or failure of the INSIDE. Specific and systematic pleiotropy can be corrected by MR-RAPS, which can provide a robust statistical estimate for our MR study even with several weak IVs (Zhao et al., 2019). The weighted median method uses the median effect of all available genetic instruments. Therefore, if at least half of the weight comes from valid instrumental variables that have no association with confounders, no horizontal pleiotropy, and a robust association with the exposure, the weighted median method could show the consistency of potential causality (Bowden et al., 2016). The weighted mode method can provide an unbiased causal relationship when the largest number of SNPs based on the similarity of causation are valid even though the remaining SNPs are invalid (Hartwig et al., 2017). Moreover, to further identify and avoid bias due to horizontal pleiotropy, we performed the MR-PRESSO method (Verbanck et al., 2018). Three components (the global test, outlier test, and distortion test) constitute the MR-PRESSO, which detects outliers, removes them, and evaluates whether there is a significant difference (*p* < 0.05) in the causal estimate after adjustment for outliers (Verbanck et al., 2018). Meanwhile, we used the Galbraith radial plot to find outliers as well (Bowden et al., 2018). Cochran’s Q test was executed to evaluate heterogeneity among variant-specific causal estimates, and a *p*-value of Cochran’s Q test of IVW > 0.05 indicated that there was no presence of heterogeneity (Bowden and Holmes, 2019). Besides, we further performed leave-one-out (LOO) analysis, forest plots, and funnel plots to evaluate the reliability and pleiotropy of the casual estimation. Scatter plots were generated to visualize the estimated effect sizes. We also applied Steiger filtering to test the causal direction of the association (Hemani et al., 2017). After removing pleiotropic SNPs, we retested the MR causal estimation. The effect estimates of IMIDs were converted to odds ratio (OR) with 95% confidence intervals (CIs), which expressed a per unit genetically determined increase in the log OR of telomere length for IMIDs. The statistically significant association of this study was determined primarily by *p* < 0.05. The statistical tests for MR were performed using the R software (version 4.1.2) with two packages, which included two-sample MR and MR-PRESSO.

### Ethical approval

All studies only utilized publicly available summary statistics. Ethical approval for these studies can be found in the original publications.

## Results

**FIGURE 2:**
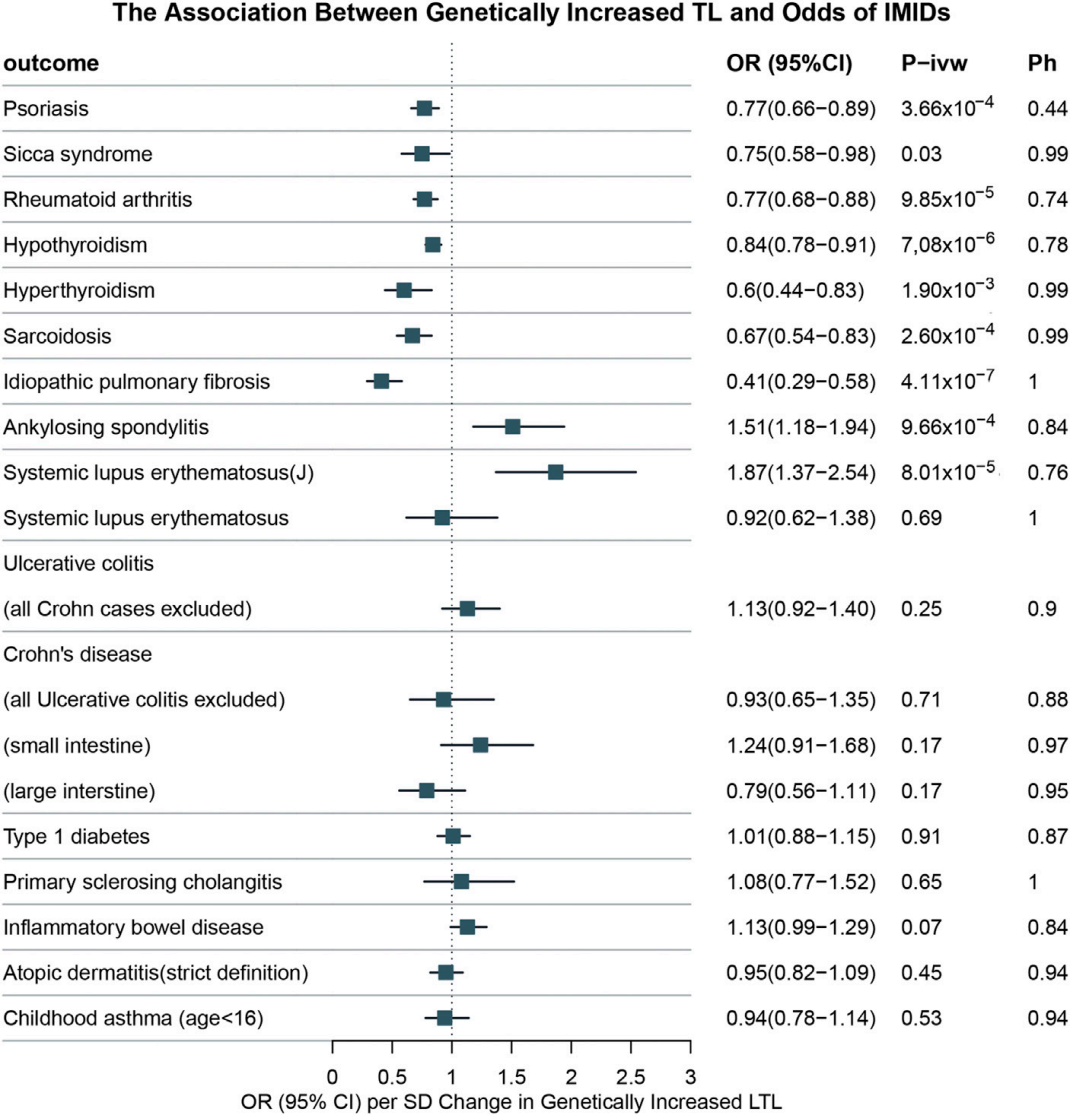
Ph, *p*-value for heterogeneity among SNPs; P-IVW, *p*-value for inverse-variance weighted (IVW). Systemic lupus erythematosus (J): a GWAS from James Bentham. Other diseases (unmarked): data from FinnGen consortium.

The results from the univariable IVW analysis of IMIDs are presented in Figure 2. Genetically determined LTL was positively related to 1 of 16 outcomes in the FinnGen biobank: AS [IVW: odds ratio = 1.51 (95% CI: 1.18–1.94) and *p* = 9.66 × 10^−4^]. The causal effects obtained from the sensitivity analysis were generally consistent. None of the outliers were revealed in MR-PRESSO. We found no evidence for the presence of pleiotropy, as indicated by the *p*-values for the MR-Egger intercept test (odds ratio = −0.002 and *p* = 0.81). Abnormality was not tested in the heterogeneity test (IVW: Q statistics = 98.29 and *p* = 0.84). The IVW showed no causal relationship between TL and SLE [odds ratio = 0.92 (95% CI: 0.62–1.38) and *p* = 0.69] in the FinnGen database. The other four MR models showed consistent results. In addition, this study did not observe potential pleiotropy (odds ratio = −0.006 and *p* = 0.53) and heterogeneity (IVW: Q statistics = 77.13 and *p* = 1.00). No outliers were revealed in MR-PRESSO as well. We found a significantly positive genetic correlation between LTL and another larger GWAS of SLE [odds ratio = 1.87 (95% CI: 1.37–2.54) and *p* = 8.01 × 10^−5^] in our MR study. Although the result of MR-Egger [odds ratio = 1.61 (95% CI: 0.89–2.89) and *p* = 0.12] was not statistically significant, it showed similarities with IVW, RAPS, weighted median, and weighted mode. No evidence for possible heterogeneity (IVW: Q statistics = 69.02 and *p* = 0.76) and horizontal pleiotropy (odds ratio = 0.005 and *p* = 0.56) was indicated. MR-PRESSO found no outliers in the MR.

LTL was significantly negatively relevant to 7 of the 16 outcomes. Our study showed a potential causal relationship between LTL and RA [IVW: odds ratio = 0.77 (95% CI: 0.68–0.88) and *p* = 9.85 × 10^−5^], hypothyroidism [IVW: odds ratio = 0.84 (95% CI: 0.78–0.91) and *p* = 7,08 × 10^−6^], hyperthyroidism [IVW: odds ratio = 0.60 (95% CI: 0.44–0.83) and *p* = 1.90 × 10^−3^], sarcoidosis [IVW: odds ratio = 0.67 (95% CI: 0.54–0.83) and *p* = 2.60 × 10^−4^], and IPF [IVW: odds ratio = 0.41 (95% CI: 0.29–0.58) and *p* = 4.11 × 10^−7^]. Despite no statistically significant results of MR-Egger [odds ratio = 0.86 (95% CI: 0.67–1.11) and *p* = 0.26] and weighted mode [odds ratio = 0.79 (95% CI: 0.61–1.04) and *p* = 0.10], our study indicated the causal effect of LTL on psoriasis [IVW: odds ratio = 0.77 (95% CI: 0.66–0.89) and *p* = 3.66 × 10^−4^]. A causal association between LTL and SS was found [IVW: odds ratio = 0.75 (95% CI: 0.58–0.98) and *p* = 0.03], even though the results of MR-Egger [odds ratio = 0.67 (95% CI: 0.43–1.06) and *p* = 0.09], weighted median [odds ratio = 0.75 (95% CI: 0.50–1.12) and *p* = 0.16], and weighted mode: [odds ratio = 0.72 (95% CI: 0.42–1.22) and *p* = 0.23] were not statistically significant. The heterogeneity test, MR-Egger intercept, and MR-PRESSO did not observe any abnormalities. The results of these are listed in Table 2. The causal direction was right by the MR Steiger test for LTL on SLE, AS, RA, hypothyroidism, hyperthyroidism, sarcoidosis, IPF, psoriasis, and SS (Supplementary Table S3).

However, LTL was not causally associated with UC [odds ratio = 1.13 (95% CI: 0.92–1.40) and *p* = 0.25], CD [odds ratio = 0.93 (95% CI: 0.65–1.35) and *p* = 0.71], T1D [odds ratio = 1.01 (95% CI: 0.88–1.15) and *p* = 0.91], PSC [odds ratio = 1.08 (95% CI: 0.77–1.52) and *p* = 0.65], IBD [odds ratio = 1.13 (95% CI: 0.99–1.29) and *p* = 0.07], AD [odds ratio = 0.95 (95% CI: 0.82–1.09) and *p* = 0.45], or childhood asthma [odds ratio = 0.94 (95% CI: 0.78–1.14) and *p* = 0.53]. MR-Egger, RAPS, weighted median, and weighted mode were in directional concordance with those of the IVW method. Furthermore, the heterogeneity test, MR-Egger intercept, and MR-PRESSO method did not show any abnormality. The detailed information is shown in Table 1.

## Discussion

Our results revealed that the variousness of LTL increased the risk of nine IMIDs, among which the risk of SLE and AS was higher in people with longer LTL. Probably due to the small sample size, no causal association between LTL and SLE was observed in the FinnGen Consortium. We therefore analyzed another GWAS of SLE (5,021 cases and 9,066 controls) and found longer LTL increased the risk of SLE. No association was observed in the causal relationship between LTL and other IMIDs.

### Comparison with previous studies

For parts of IMIDs, our findings were similar to those from some previous studies, while for other IMIDs, they were the opposite. The results from the MR study based on data from the FinnGen Biobank corroborate the findings of retrospective studies that show shorter LTL is a risk factor for RA (Blinova et al., 2016; Gamal et al., 2018; Lee and Bae, 2018), SS (Kawashima et al., 2011; Noll et al., 2020), sarcoidosis (Maeda et al., 2009; Afshar et al., 2021), IPF (McDonough et al., 2018; Ley et al., 2020), and psoriasis (Liu et al., 2008). Furthermore, our results are also similar to previously reported MR studies of LTL and the risk of RA (Zeng et al., 2020) and IPF (Duckworth et al., 2021). Meanwhile, our results are in accordance with previous studies, which found that longer LTL is likely to increase the risk of AS (Tamayo et al., 2010), and found a weak or null correlation between childhood asthma (Belsky et al., 2014; Suh et al., 2019a), IBD (which includes UC and CD) (Getliffe et al., 2005), and LTL. Interestingly, Tamayo et al. (2010) implemented a cross-sectional study in 2010 and found longer telomeres in patients with AS (N case = 59) in PBMCs than in 130 controls, but found that a shorter average TL with aging was evident in AS 4 years thereafter (Tamayo et al., 2014). According to a case–control study, the extent of telomere loss due to aging was almost consistent in UC, CD, and control patients (Getliffe et al., 2005). Nevertheless, Risques et al. (2008) found that the TL was shorter in patients with UC than in control participants, and notably, the TL of colon stromal cells was not affected. Thus, the role of LTL in IMIDs requires further study. However, the MR study found different results from previous studies: shorter or longer LTL may increase the risk of T1D (Sanchez et al., 2020; Svikle et al., 2022), AD (Wu et al., 2000), and PSC (Laish et al., 2015). For instance, two cross-sectional studies found that the TL was shorter in T1D patients than in non-diabetic subjects (Sanchez et al., 2020; Syreeni et al., 2022). A longitudinal study suggested that telomeres shorten in 55.3% of subjects with T1D and further reported that shorter TL had an adverse effect on cardiometabolic and renal profiles (Syreeni et al., 2022). Observational studies specializing in LTL with relation to the risk of hypothyroidism or hyperthyroidism are scarce. We retrieved an analysis of this aspect from a previous study (Codd et al., 2021). It investigated differences in LTL and thyroid function (hypothyroidism and hyperthyroidism) between MR and an observational study. It found limited evidence of causality between LTL and hyperthyroidism in the observational study but not in the MR study. However, it evidenced a significant LTL shortening in subjects with hypothyroidism *via* MR and a previous observational study which supports our findings (Codd et al., 2021). The results of the retrospective studies are mixed, or previous findings are inconsistent with MR, which may be interfered with by confounding factors or reverse causation, whereby changes in telomere length arise as a result of disease.

### Potential mechanisms

The mechanisms involving IMIDs are numerous, are complicated, and remain unclear. These diseases, despite their specific characteristics, have certain overlapping aspects. We hypothesized several possible explanations for why the shorter LTL is likely to be a risk factor for multiple IMIDs in our results. Several studies have shown that shortened LTL produces increased secretion of inflammatory cytokines/chemokines such as IL-1, IL-12, IL-17, IFN-γ, TNF-α, and IL-6, which could contribute to the imbalance of pro- and anti-inflammatory cytokines, resulting in corresponding symptoms (Chizzolini et al., 2009; Kunz and Ibrahim, 2009; Rodier et al., 2009; O'Donovan et al., 2011). People with short TL due to telomerase mutation might have severe immunodeficiency of T cells producing pro-inflammatory cytokines, which in turn lead to producing corresponding symptoms (Maly and Schirmer, 2015; Wagner et al., 2018). A study suggested telomerase reverse transcriptase was a previously unrecognized NF-κB target gene. In other words, the inhibition of a master regulator of inflammation (NF-κB) may abolish the induction of telomerase transcription (Gizard et al., 2011) and then cause LTL to shorten.

Our findings revealed that TL is genetically associated with SLE and AS, which may indicate a potentially similar mechanism between the two IMIDs. Notably, a longer TL is not correlated with younger biological age in comparison to controls. A few possibilities can be interpreted as follows. First, Calado et al. (2009)observed that in bone marrow CD34^+^ cells, androgen and estradiol could stimulate telomerase activity, which plays an important role in maintaining the length of telomeres; moreover, androgen also mediated telomerase activity in both normal peripheral blood lymphocytes. Their findings may indicate a significant difference between males and females in the incidence of SLE (Tian et al., 2022) and AS (Tarannum et al., 2022; Zhang et al., 2022). The antigen presentation process stimulates telomerase in normal B cells and T cells, leading to increased telomerase activity (Igarashi and Sakaguchi, 1997; Hathcock et al., 2005). Upregulation of telomerase activity may partially compensate for the shortened telomeres, and this compensation would allow the capacity of replication of the responding peripheral blood lymphocytes to be maintained or even prolonged. Therefore, during the immune response, the function of these lymphocytes is enhanced and mediates the onset of IMIDs (Hathcock et al., 2005). In addition, TRF1 and POT1, two shelterin subunits, are regulators that control telomere elongation (de Lange, 2005; Okamoto et al., 2008), whereby they may be involved in the mechanism by which longer LTL causes SLE and AS.

Numerous studies have reported that many lifestyle habits involved with IMIDs have a suspiciously intimate correlation with the alteration of the TL. For instance, chronic stress (Shalev et al., 2013), dietary habits (Crous-Bou et al., 2014; Buttet et al., 2022; Gong et al., 2022) such as a Mediterranean diet (Crous-Bou et al., 2014), exercise (Latifovic et al., 2016; Dankel et al., 2017; Puterman et al., 2018; Buttet et al., 2022), alcohol consumption (Pavanello et al., 2011; Martins de Carvalho et al., 2019), and smoking (Huzen et al., 2014; Müezzinler et al., 2015; Latifovic et al., 2016) exert significant effects on the pathophysiology of telomere dynamics, but the results of researching their impact on causality is unreliable due to the deficiency of designs. Furthermore, LTL was measured in 1,156 individuals in four longitudinal studies by Benetos et al. (2013). The reality is that over time, LTL is fixed at a specific level. Therefore, lifestyle habits and their modification during adulthood have little impact on LTL ranking.

### Clinical implications

Our findings can give certain suggestions on the implications of LTL in clinical works. Since LTL is a causal risk factor for IMIDs, it can be used as a predictor of risk of IMIDs. Although changes in LTL show no relationship with IMIDs, it can be used as a target to further discover the mechanism of IMIDs, which may provide new diagnostic biomarkers and potential treatment targets.

### Strength and limitations

The multiple advantages of this MR study are as follows. First, the robust SNPs for LTL are identified from the largest and latest GWAS. Second, to reduce population stratification bias as much as possible, most of the samples in this study were from the FinnGen consortium, and the other GWAS, although not part of the Finnish database, was still from the European population. Third, the estimation of the SNPs associated with LTL and IMIDs was obtained from two independent subjects in order to reduce bias in the evaluation of causality. Moreover, we selected robust SNPs *via* stringent quality control conditions and employed comprehensive analysis methodologies. Therefore, MR might be a useful tool for investigating the potential causality in a scientific context.

This study is also subject to some limitations. First, we only conducted analyses for peripheral leukocyte TL (Codd et al., 2021) and not for TL of specific tissues directly affected by each IMID, although it is highly correlated with TL in other tissues (Demanelis et al., 2020). Second, the possibility that the LTL-related SNPs affect IMID outcomes through other causal pathways than through LTL exposure cannot be completely ruled out. Third, our results may not fully represent IMIDs (since not all studies of IMIDs provided publicly available data, and our results are underpowered to conclude the causality regarding secondary disease outcomes). Fourth, while the sample size is large in the FinnGen Biobank, too small numbers of some IMID cases lead to unbalanced frequency matching of case and control groups. Then, this may affect the accuracy and precision of the analytical results. Fifth, our MR analysis hypothesized a linear shape of the association between LTL and IMIDs, although the possibility of other associational shapes such as “J” and “U” should be taken into account. Sixth, we failed to explore the statistical differences in gender regarding the causal effect between LTL and IMIDs (Pierce and Burgess, 2013) since details of personal data in the FinnGen biobank and the GWAS data set are not published. Seventh, since all the individuals involved in our study were exclusively of European descent, our findings might not fully represent whole populations. Thus, it is not plausible to extrapolate the findings from our results to other populations. Eighth, our analysis can yield reliable results when three assumptions are satisfied (Figure 1). However, the second and third assumptions are difficult to test in many cases, and they are also difficult to satisfy. Therefore, we need to treat the results of MR with caution.

## Conclusion

In summary, the role of LTL varies in different IMIDs. Owing to the different pathogenic factors of IMIDs, TL and its potential effect might be various. Further research is required to further discover the mechanism and causes of change in TL in order to reveal the initial pathogenic factors of IMIDs. Only when the mechanism or causes is fully understood, scientists can optimize therapeutic interventions and attempt to extend human life span.

## Data Availability

The original contributions presented in the study are included in the article/Supplementary Material; further inquiries can be directed to the corresponding author.
