# Supplementary material for: Immune-mediated inflammatory diseases and leukocyte telomere length: A Mendelian randomization study
Source: Front Genet. 2023 Apr 17;14:1129247. doi: 10.3389/fgene.2023.1129247 (PMC10150136; doi:10.3389/fgene.2023.1129247)
Supplement: Supplementary file 8 [file DataSheet1.PDF]

Supplementary Materials

Supplementary Table1. SNPs used for the LTL instruments in the Mendelian Randomization analyses.

Supplementary Table 2. GWAS summary statistics on outcomes: source and description.

Supplementary Table 3 MR steiger analysis showing the causal direction of leukocyte telomere length (LTL) on Immune-mediated Inflammatory Diseases (IMIDs).

Supplementary Table1. SNPs used for the LTL instruments in the Mendelian Randomization analyses.

SNP	effect_allele	other_allele	eaf	beta	se	F	pval
rs2977608	A	C	0.256	-0.013	0.002	30.694	3.00E-08
rs66731853	G	A	0.683	0.018	0.002	68.115	1.50E-16
rs6669563	G	A	0.562	-0.018	0.002	81.115	2.10E-19
rs4660456	A	G	0.770	-0.014	0.002	35.307	2.80E-09
rs41269079	T	A	0.811	-0.015	0.003	36.294	1.70E-09
rs139795227	A	C	0.986	-0.060	0.009	47.112	6.70E-12
rs145114957	C	G	0.957	-0.027	0.005	29.860	4.60E-08
rs4498805	G	T	0.453	-0.015	0.002	56.489	5.70E-14
rs17464525	G	A	0.819	0.032	0.003	156.506	6.60E-36
rs11579626	A	C	0.915	-0.027	0.004	54.916	1.30E-13
rs6587577	A	G	0.174	0.018	0.003	47.752	4.80E-12
rs6659669	C	T	0.395	0.012	0.002	32.571	1.10E-08
rs932002	C	T	0.849	0.040	0.003	206.672	7.30E-47
rs2555104	A	C	0.566	0.014	0.002	47.139	6.60E-12
rs56178008	T	A	0.563	-0.014	0.002	50.904	9.70E-13
rs6751209	T	C	0.796	0.014	0.002	31.960	1.60E-08
rs965109	C	T	0.976	0.102	0.006	246.173	1.80E-55
rs188918174	C	T	0.964	-0.040	0.005	54.977	1.20E-13
rs12619538	A	C	0.859	-0.017	0.003	33.865	5.90E-09
rs17803849	C	T	0.595	-0.027	0.002	180.268	4.20E-41
rs4535042	T	G	0.701	-0.012	0.002	30.532	3.30E-08
rs869785	T	C	0.328	0.015	0.002	47.915	4.40E-12
rs2230590	T	C	0.489	0.016	0.002	61.927	3.60E-15
rs78491606	A	C	0.982	0.076	0.007	104.128	1.90E-24
rs13062095	T	C	0.672	-0.014	0.002	41.874	9.70E-11
rs6776756	G	A	0.402	0.017	0.002	73.300	1.10E-17
rs9878436	C	T	0.566	0.014	0.002	50.491	1.20E-12
rs10936598	A	C	0.676	0.066	0.002	935.118	1.00E-200
rs76094497	G	A	0.948	-0.028	0.005	36.430	1.60E-09
rs563069803	G	T	0.982	0.057	0.009	43.873	3.50E-11
rs112394943	T	C	0.837	0.020	0.003	49.905	1.60E-12
rs2282764	A	G	0.858	0.022	0.003	60.039	9.30E-15
rs871134	C	T	0.431	0.018	0.002	81.551	1.70E-19
rs10805346	T	C	0.561	-0.012	0.002	33.541	7.00E-09
rs4695407	A	G	0.492	-0.014	0.002	50.101	1.50E-12
rs10024820	T	C	0.611	0.014	0.002	49.366	2.10E-12
rs4435700	C	A	0.235	-0.054	0.002	507.529	2.20E-112
rs11746381	T	C	0.943	-0.033	0.004	58.598	1.90E-14
rs11745132	G	T	0.992	-0.071	0.012	35.284	2.80E-09
rs7726159	C	A	0.673	-0.071	0.002	1105.852	1.00E-200
rs61748181	C	T	0.971	0.059	0.006	98.800	2.80E-23
rs6881568	C	A	0.637	-0.017	0.002	66.385	3.70E-16
rs6873104	A	T	0.899	0.025	0.003	53.496	2.60E-13
rs55747751	G	A	0.923	0.021	0.004	31.815	1.70E-08
rs185174247	G	A	0.944	-0.037	0.004	73.400	1.10E-17
rs80324517	G	A	0.952	-0.040	0.005	72.313	1.80E-17
rs7772289	G	T	0.497	-0.018	0.002	76.990	1.70E-18
rs2763979	C	T	0.640	0.028	0.002	178.107	1.30E-40
rs112817717	G	C	0.524	-0.014	0.002	41.206	1.40E-10
rs141127771	G	A	0.741	0.016	0.003	35.618	2.40E-09
rs9398196	A	G	0.480	0.014	0.002	50.942	9.50E-13
rs61405042	C	T	0.971	0.050	0.006	69.282	8.50E-17
rs13230646	T	C	0.751	0.017	0.002	55.603	8.90E-14
rs11769630	T	A	0.928	0.026	0.004	43.476	4.30E-11
rs38664	T	C	0.403	0.012	0.002	35.197	3.00E-09
rs2056726	G	A	0.786	0.023	0.002	87.635	7.90E-21
rs7790856	C	T	0.711	0.044	0.002	393.042	1.80E-87
rs565783711	C	T	0.982	-0.057	0.008	49.655	1.80E-12
rs6969930	T	C	0.387	0.020	0.002	100.112	1.40E-23
rs117407747	C	T	0.972	-0.045	0.006	54.246	1.80E-13
rs1985369	A	G	0.132	0.031	0.003	107.403	3.60E-25

rs2306646	G	C	0.441	0.021	0.002	107.587	3.30E-25
rs762679	T	A	0.143	-0.031	0.003	118.373	1.40E-27
rs10808899	G	C	0.111	0.030	0.003	89.464	3.10E-21
rs10112752	G	A	0.570	0.029	0.002	201.565	9.50E-46
rs1023767	G	A	0.762	0.018	0.002	61.246	5.00E-15
rs4742448	C	G	0.531	-0.015	0.002	56.239	6.40E-14
rs11557154	C	T	0.870	0.034	0.003	132.559	1.10E-30
rs4743037	C	T	0.769	-0.015	0.002	38.624	5.10E-10
rs10905255	G	T	0.421	0.018	0.002	80.738	2.60E-19
rs7906139	G	A	0.870	0.019	0.003	39.214	3.80E-10
rs12412214	G	A	0.720	0.025	0.002	121.218	3.40E-28
rs11190270	G	A	0.798	0.014	0.003	29.790	4.80E-08
rs9419958	T	C	0.139	0.081	0.003	760.033	2.60E-167
rs805664	A	T	0.013	0.087	0.009	86.197	1.60E-20
rs77231040	G	C	0.994	-0.099	0.013	53.982	2.00E-13
rs939916	G	A	0.330	-0.024	0.002	124.475	6.60E-29
rs1609812	G	A	0.160	-0.047	0.003	290.464	3.90E-65
rs10840270	C	G	0.344	-0.014	0.002	45.815	1.30E-11
rs2293579	G	A	0.614	0.013	0.002	39.504	3.30E-10
rs611646	T	A	0.591	0.037	0.002	327.412	3.50E-73
rs11212631	T	C	0.801	0.019	0.003	56.861	4.70E-14
rs6590343	A	G	0.484	-0.012	0.002	36.522	1.50E-09
rs10845387	G	A	0.647	0.014	0.002	45.480	1.50E-11
rs12369950	T	C	0.859	0.018	0.003	37.751	8.00E-10
rs79755767	G	A	0.904	-0.028	0.003	65.895	4.80E-16
rs17445108	G	A	0.873	0.017	0.003	31.498	2.00E-08
rs1907702	G	A	0.233	-0.015	0.002	38.340	5.90E-10
rs10774624	G	A	0.467	-0.015	0.002	53.243	2.90E-13
rs76666449	T	C	0.899	-0.030	0.003	78.458	8.20E-19
rs10773176	A	G	0.259	0.017	0.002	56.650	5.20E-14
rs28577594	G	C	0.290	-0.019	0.002	70.168	5.40E-17
rs1332941	A	G	0.180	-0.026	0.003	88.210	5.90E-21
rs670180	T	A	0.431	0.012	0.002	32.521	1.20E-08
rs9600019	C	T	0.664	-0.013	0.002	35.594	2.40E-09
rs3093888	G	A	0.949	0.029	0.005	41.004	1.50E-10
rs73581419	C	T	0.893	-0.023	0.003	50.273	1.30E-12
rs113525195	C	A	0.710	0.012	0.002	30.645	3.10E-08
rs45604339	C	T	0.658	0.020	0.002	93.394	4.30E-22
rs137901416	G	A	0.900	-0.046	0.003	189.238	4.70E-43
rs8006485	G	T	0.547	-0.019	0.002	91.418	1.20E-21
rs1957937	A	T	0.840	-0.021	0.003	58.659	1.90E-14
rs11629678	G	A	0.671	-0.022	0.002	104.991	1.20E-24
rs181647350	T	C	0.761	0.034	0.002	201.990	7.70E-46
rs1980240	A	C	0.595	-0.013	0.002	40.312	2.20E-10
rs5742915	T	C	0.554	-0.019	0.002	90.846	1.60E-21
rs113490934	T	C	0.938	0.035	0.004	71.229	3.20E-17
rs12932179	A	G	0.439	0.014	0.002	45.160	1.80E-11
rs182059586	T	C	0.975	0.057	0.007	70.373	4.90E-17
rs450962	A	G	0.716	-0.014	0.002	33.870	5.90E-09
rs1105407	C	G	0.833	-0.016	0.003	34.099	5.20E-09
rs76219171	G	A	0.942	-0.036	0.004	69.466	7.80E-17
rs528301822	A	T	0.711	-0.024	0.002	118.080	1.70E-27
rs62050964	G	A	0.662	0.019	0.002	77.106	1.60E-18
rs11866592	G	A	0.858	-0.035	0.003	146.073	1.30E-33
rs2303262	C	T	0.223	0.047	0.002	382.932	2.90E-85
rs11117354	T	C	0.303	-0.023	0.002	112.098	3.40E-26
rs12925933	A	C	0.338	0.015	0.002	47.033	7.00E-12
rs59409453	A	G	0.269	-0.020	0.002	77.118	1.60E-18
rs12451892	T	C	0.619	0.012	0.002	31.307	2.20E-08
rs4724	G	A	0.883	0.055	0.003	307.006	9.80E-69
rs75664430	C	G	0.752	0.024	0.002	102.883	3.60E-24
rs111527438	T	C	0.649	-0.013	0.002	35.090	3.10E-09

rs12941945	A	G	0.832	0.026	0.003	94.099	3.00E-22
rs7209057	G	A	0.439	-0.012	0.002	33.943	5.70E-09
rs6501181	T	A	0.463	-0.013	0.002	42.685	6.40E-11
rs144204502	C	T	0.987	0.101	0.009	121.249	3.40E-28
rs3891167	A	G	0.747	0.043	0.002	315.777	1.20E-70
rs150150565	C	T	0.979	-0.064	0.007	74.269	6.80E-18
rs116863223	G	A	0.988	0.082	0.009	76.164	2.60E-18
rs139669835	C	T	0.991	0.061	0.011	33.812	6.10E-09
rs16978028	A	T	0.856	0.030	0.003	110.345	8.20E-26
rs2276182	C	G	0.597	-0.023	0.002	130.728	2.80E-30
rs1879100	C	T	0.138	0.019	0.003	42.234	8.10E-11
rs11085072	C	T	0.763	0.013	0.002	31.005	2.60E-08
rs8105767	A	G	0.705	-0.033	0.002	222.565	2.50E-50
rs4530278	G	T	0.402	-0.014	0.002	45.540	1.50E-11
rs429358	T	C	0.846	-0.017	0.003	39.205	3.80E-10
rs8102497	G	A	0.568	0.015	0.002	54.709	1.40E-13
rs6054257	G	A	0.206	0.014	0.002	32.711	1.10E-08
rs1291143	A	C	0.151	-0.049	0.003	310.393	1.80E-69
rs188546415	G	A	0.984	0.054	0.009	36.807	1.30E-09
rs3865523	G	T	0.188	0.055	0.003	420.626	1.80E-93
rs552374163	C	T	0.992	0.072	0.012	36.398	1.60E-09
rs35640778	G	A	0.979	0.209	0.007	886.250	9.60E-195
rs61736615	G	A	0.963	0.037	0.005	47.866	4.60E-12
rs115610405	C	A	0.981	0.110	0.007	223.197	1.80E-50
rs139228302	G	A	0.975	0.047	0.007	52.733	3.80E-13
rs151255005	C	A	0.987	-0.054	0.009	37.936	7.30E-10
rs28502153	C	A	0.622	0.022	0.002	109.637	1.20E-25
rs6007020	T	C	0.632	-0.014	0.002	47.778	4.80E-12
rs131795	A	T	0.208	0.025	0.002	101.438	7.40E-24
rs1003322	C	A	0.786	-0.014	0.002	32.782	1.00E-08

eaf, effect allele frequency; SNP, single-nucleotide polymorphisms.

Supplementary Table 2. GWAS summary statistics on outcomes: source and description.

Outcomes	Population	Cases	Controls	phenocode	PMID/ consortium
systemic lupus erythematosus	European	835	300162	M13_SLE	FinnGen
Ulcerative colitis (all Crohn cases excluded)	European	3407	303191	K11_UC_NOCD	FinnGen
Crohn disease (all Ulcerative Colitis excluded)	European	1021	301234	K11_CD_NOUC	FinnGen
Crohn disease (small intestine)	European	1501	296008	CHRONSMALL	FinnGen
Crohn disease (large intestine)	European	1220	296008	CHRONLARGE	FinnGen
type 1 diabetes	European	8671	255466	E4_DM1	FinnGen
Primary sclerosing cholangitis	European	1231	273442	K11_CHOLANGI	FinnGen
inflammatory bowel disease	European	8704	300450	K11_IBD	FinnGen
atopic dermatitis (strict definition)	European	7895	300873	ATOPIC_STRICT	FinnGen
childhood asthma (age<16)	European	4549	175182	ASTHMA_CHILD	FinnGen
ankylosing spondylitis	European	2252	227388	M13_ANKYLOSPON	FinnGen
psoriasis	European	6995	299128	L12_PSORIASIS	FinnGen
sicca syndrome	European	1981	300162	M13_SJOGREN	FinnGen
rheumatoid arthritis	European	9855	202617	M13_RHEUMA	FinnGen
hypothyroidism	European	33422	227415	E4_HYTHY_AI_STRICT	FinnGen
hyperthyroidism	European	1421	231654	AUTOIMMUNE_HYPERTHYROIDISM	FinnGen
sarcoidosis	European	3103	304494	D3_SARCOIDOSIS	FinnGen
idiopathic pulmonary fibrosis	European	1514	306063	IPF	FinnGen
SLE (James Bentham)	European	5201	9066	No phenocode.<br>diagnostic criteria:<br>Standard American College of Rheumatology classification criteria	26502338

Supplementary Table 3 MR steiger analysis showing the causal direction of leukocyte telomere length (LTL) on Immune-mediated Inflammatory Diseases (IMIDs).

Exposure	Outcomes	Correct causal direction	r2 exposure	r2 outcome	Steiger P-value
**leukocyte telomere length (LTL)**	**ankylosing spondylitis**	**TRUE**	2.86×10^{-2}	$4.75 \text{ x } 10^{-4}$	<0.0001
	**psoriasis**	**TRUE**	2.67×10^{-2}	$3.83 \text{ x } 10^{-4}$	<0.0001
	**sicca syndrome**	**TRUE**	2.84×10^{-2}	$2.71 \text{ x } 10^{-4}$	<0.0001
	**rheumatoid arthritis**	**TRUE**	2.55×10^{-2}	$5.15 \text{ x } 10^{-4}$	<0.0001
	**hypothyroidism**	**TRUE**	2.61×10^{-2}	$3.96 \text{ x } 10^{-4}$	<0.0001
	**hyperthyroidism**	**TRUE**	2.67×10^{-2}	$3.67 \text{ x } 10^{-4}$	<0.0001
	**sarcoidosis**	**TRUE**	2.70×10^{-2}	$2.87 \text{ x } 10^{-4}$	<0.0001
	**idiopathic pulmonary fibrosis**	**TRUE**	2.28×10^{-2}	$2.98 \text{ x } 10^{-4}$	<0.0001
	**SLE (James Bentham)**	**TRUE**	1.90×10^{-2}	$5.93 \text{ x } 10^{-3}$	$3.70 \text{ x } 10^{-13}$
